# Synthesis reveals approximately balanced biotic differentiation and homogenization

**DOI:** 10.1126/sciadv.adj9395

**Published:** 2024-02-21

**Authors:** Shane A. Blowes, Brian McGill, Viviana Brambilla, Cher F. Y. Chow, Thore Engel, Ada Fontrodona-Eslava, Inês S. Martins, Daniel McGlinn, Faye Moyes, Alban Sagouis, Hideyasu Shimadzu, Roel van Klink, Wu-Bing Xu, Nicholas J. Gotelli, Anne Magurran, Maria Dornelas, Jonathan M. Chase

**Affiliations:** ^1^ German Centre for Integrative Biodiversity Research (iDiv) Halle-Jena-Leipzig, Leipzig, Germany.; ^2^ Department of Computer Science, Martin Luther University Halle-Wittenberg, Halle (Saale), Germany.; ^3^ School of Biology and Ecology and Mitchell Center for Sustainability Solutions, University of Maine, Orono, ME, USA.; ^4^ Centre for Biological Diversity, School of Biology, University of St Andrews, St Andrews, Scotland.; ^5^ Guia Marine Lab, MARE, Faculty of Sciences, University of Lisbon, Lisbon, Portugal.; ^6^ Institute of Biodiversity, Friedrich Schiller University Jena, Dornburger Straße 159, 07743 Jena, Germany.; ^7^ Department of Ecosystem Services, Helmholtz Centre for Environmental Research - UFZ, Permoserstr. 15, 04318 Leipzig, Germany.; ^8^ Leverhulme Centre for Anthropocene Biodiversity, Berrick Saul Second Floor, University of York, York, UK.; ^9^ Department of Biology, College of Charleston, Charleston, SC, USA.; ^10^ Department of Mathematical Sciences, Loughborough University, Leicestershire, UK.; ^11^ Department of Data Science, Kitasato University, Kanagawa, Japan.; ^12^ Department of Biology, University of Vermont, Burlington, VT, USA.

## Abstract

It is commonly thought that the biodiversity crisis includes widespread declines in the spatial variation of species composition, called biotic homogenization. Using a typology relating homogenization and differentiation to local and regional diversity changes, we synthesize patterns across 461 metacommunities surveyed for 10 to 91 years, and 64 species checklists (13 to 500+ years). Across all datasets, we found that no change was the most common outcome, but with many instances of homogenization and differentiation. A weak homogenizing trend of a 0.3% increase in species shared among communities/year on average was driven by increased numbers of widespread (high occupancy) species and strongly associated with checklist data that have longer durations and large spatial scales. At smaller spatial and temporal scales, we show that homogenization and differentiation can be driven by changes in the number and spatial distributions of both rare and common species. The multiscale perspective introduced here can help identify scale-dependent drivers underpinning biotic differentiation and homogenization.

## Teaser

Homogenization is most common at large temporal and spatial scales but is balanced by differentiation at smaller scales.

## INTRODUCTION

With an ever-growing human footprint (*1*), Earth’s biodiversity is inevitably changing (*2*). There is substantial evidence that humans are accelerating the global extinction rate (*3*). However, at local scales, widespread changes in species composition are accompanied by little evidence for a strong overall directional trend in species richness amidst substantial variability (*4*–*7*) [albeit with some controversy; (*8*)]. One common explanation for the perceived discrepancy between declining global diversity and little to no change in the number of species found at local scales is biotic homogenization (*9*–*11*).

Biotic homogenization occurs when spatially distinct locations become more similar to one another in species composition through time (*12*–*14*). Two opposing forces can lead to homogenization. First, there can be increases in the numbers of widespread (high occupancy) species, such as native species that benefit from changing landscapes, or non-native species expanding their range. This can lead to biotic homogenization among local communities even with increasing diversity at smaller and larger scales. Second, there can be extirpation of rare (low occupancy) species from regions. Such extirpations are often accelerated when changing landscapes disfavor rare species’ habitats or population growth. However, landscapes can also become more heterogeneous (*15*), which can lead to biotic differentiation. Here, different species are favored in different habitat types, leading to lower similarity among local communities through time. Differentiation can occur when non-native species are introduced locally, but do not become widespread, or when formerly widespread species are locally extirpated (*16*, *17*). While both biotic homogenization and differentiation are expected theoretically under different scenarios, and frequently observed, a synthesis of just how frequently and intensely they occur in surveys of biodiversity change through time is lacking. Moreover, despite the intrinsic connection between changes in diversity across scales and the homogenization and differentiation of community composition through time (*16*, *17*), the empirical relationships among these have not been well quantified and synthesized.

**Fig. 1.**
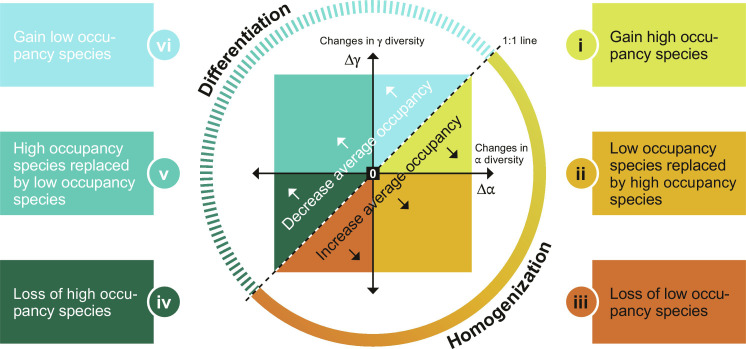
Mechanisms that underpin changes in spatial variation of species composition can be understood by examining the relationship between changes in regional- and local-scale species richness through time. When richness changes at regional (∆γ) and local (∆α) scales are calculated as proportional changes (i.e., on a log scale), assemblages below the dashed 1:1 line, i.e., ∆γ < ∆α, are being homogenized, β diversity is decreasing, and average occupancy is increasing. Conversely, assemblages above the diagonal dashed 1:1 line, i.e., ∆γ > ∆α, are differentiating, β diversity is increasing, and average occupancy is decreasing. Homogenization can be further characterized as being due to the following: (i) increased numbers of species with high occupancy (i.e., that occupy the majority of sites in the region); (ii) species with low occupancy (i.e., occupy few sites in the region) being replaced by those with high occupancy; (iii) the number of species with low occupancy is decreasing. Similarly, differentiation can be due to the following: (iv) The number of species with high occupancy is decreasing; (v) species with low occupancy are replacing those with high occupancy; (vi) the number of species with low occupancy is increasing.

Whittaker’s (*18*) diversity partition presents a parsimonious framework to examine the relationship between rates of change across spatial scales and the homogenization or differentiation of community composition. The diversity of a single site (e.g., a local site) is α diversity, and the combined diversity of several local sites (e.g., a region) is γ diversity. Variation in species composition among local sites, referred to as β diversity, is given by: $\beta =\gamma /\bar{\alpha }$ (where $\bar{\alpha }$ is the average local diversity across sites in a region). Therefore, if rates of change in α and γ diversity through time are not equal, then β diversity is changing too. Moreover, these changes in β diversity can be mathematically linked to changes in the number of sites species occupy (i.e., occupancy). Average occupancy ( $\bar{o}$ ), or the proportion of sites within a region occupied by species *i* (o*_i_*), is related to Whittaker’s formula by $\beta =\gamma /\bar{\alpha }=\gamma /\left(\Sigma o_{i}\right)=\gamma /\left(\gamma \bar{o}\right)=1/\bar{o}$ (*19*). Regardless of whether β diversity decreases (homogenization) or increases through time (differentiation), with this framework we can make direct links to changes in diversity at two different scales, and to changes in the average proportion of sites that species occupy (Fig. 1).

We can gain a more ecologically informed understanding of scale-dependent biodiversity change by evaluating change in α, β, and γ diversity simultaneously. We illustrate this more detailed picture of scale-dependent change in Fig. 1, showing six qualitatively distinct scenarios that emerge in the intersecting space of temporal changes in α and γ diversity. The 1:1 line (i.e., ∆γ = ∆α, ∆β = 0) represents equal changes at both scales and delineates the boundary between homogenization and differentiation (Fig. 1). When ∆α > ∆γ and a region is below the 1:1 line, homogenization occurs because species have an increased average occupancy. Here, three of the six scenarios are possible. (i) Increased numbers of high occupancy species, for example, due to environmental changes that favor widespread, generalist and/or non-native species, could drive increases in α diversity proportionately more than γ diversity, leading to homogenization (with ∆α > ∆γ > 0; Fig. 1i). (ii) If widespread species replace low occupancy species, then average occupancy increases and homogenization is associated with diversity gains at the α scale and losses at the γ scale (∆α > 0, ∆γ < 0; Fig. 1ii). (iii) If low occupancy species (e.g., endemic species restricted to few sites) are regionally extirpated, then homogenization is associated with diversity loss at both the α and γ scales (∆γ < ∆α < 0; Fig. 1iii). These distinct scenarios all describe cases of biotic homogenization where β diversity declines through time, but variation in the nature of scale-dependent change at the α diversity and γ diversity scales could have different implications for understanding biodiversity change, as well as how to mitigate it via conservation policy. Three further parallel and distinct scenarios of differentiation (and decreasing average occupancy), leading to increasing β diversity through time, are also possible. (iv) Fewer widespread or high occupancy species could, for example, result from increased habitat heterogeneity, and greater losses at α relative to γ scales combine to increase β diversity (∆α < ∆γ < 0; Fig. 1iv). Increased habitat heterogeneity could also lead to increases in γ diversity, accompanied by either (v) α diversity declines if low occupancy species replace high occupancy species (∆α < ∆γ, ∆α < 0, ∆γ > 0; Fig. 1v) or (vi) increased α diversity due to increased numbers of low occupancy species (∆γ > ∆α > 0; Fig. 1vi; see fig. S1 for further illustration of scale-dependent variation).

To assess overarching trends in scale-dependent biodiversity change from observational time series, we use a compilation of 525 datasets (fig. S2) documenting taxonomic diversity through time across spatial scales and the typology of Fig. 1 to estimate changes in α, β, and γ diversity. Among these datasets, there were 461 studies where we could calculate sample effort-controlled species richness from local sites (α diversity) and from the broader region (γ diversity). To this, we added 64 datasets compiled from presence-absence species “checklists”; these data typically encompass much longer time spans, documenting historical species composition before major human disruption and a more contemporary time period, but most frequently only have two time points. We include species checklist data despite their coarse nature as they are typically relatively complete species inventories and have been used to make key contributions to our understanding of long-term trends in introductions and extinctions (*20*, *21*), as well as biotic homogenization (*22*, *23*). For all datasets, we used a minimum of at least four sites per region and at least 10 years between the first and last samples. At the α scale, spatial grains varied from <1 m^2^ (e.g., plant quadrats or pitfall traps) to checklists of grid cells or whole countries or islands. At the γ scale, spatial extents ranged from several samples within a field site (<<1 km^2^) to species checklists on islands distributed across several oceans.

We examine scale-dependent biodiversity change with two complementary analyses. First, to examine whether there were any general tendencies among all 525 datasets (many of which only had two time points), we calculated temporal changes in diversity at the smaller, α scale, and a larger, γ scale, taken as the sum of species in all of the local samples combined. Rates of change were calculated at each scale as a log ratio using samples at the start and end of each time series standardized by the duration between samples {i.e., log[(*S*_t2_/*S*_t1_)/(t2 − t1 + 1)], where *S* is species richness, and t1 and t2 denote the year of first and last sample, respectively}. We fit multilevel models separately to data at the α and γ scales; both models adjusted for residual heterogeneity associated with duration and the different data sources (i.e., resurveys and checklists; see Material and Methods). We then used a subset of the datasets where multiple time points were available and relative abundances were sampled (*n* = 229, median duration = 16 years, range = 10 to 72 years; see Material and Methods) to determine the influence of (i) calculating rates of change using only two time points and (ii) rare species on our results. Specifically, we estimated rates of change in species richness and a diversity metric less sensitive to rare species [the effective numbers of species conversion of Simpson’s concentration; (*24*)] using linear models fit to time series.

## RESULTS AND DISCUSSION

**Fig. 2.**
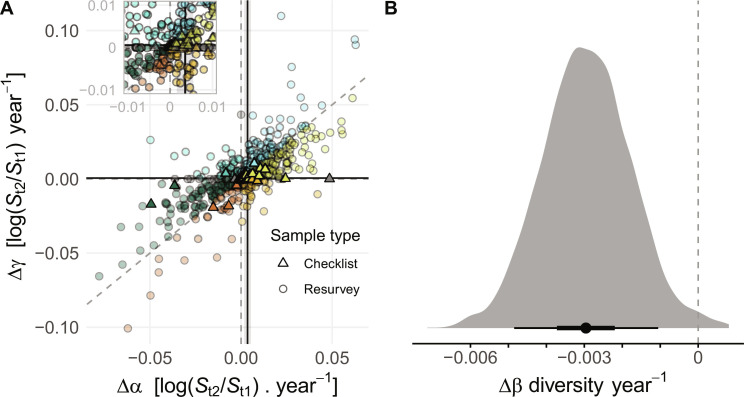
Patterns of homogenization and differentiation across 461 metacommunities and 64 checklists. (**A**) Empirical estimates of γ scale changes (∆γ) as a function of α scale changes (∆α), both axes show log ratios standardized by the number of years between the estimates; black lines show average γ and α scale rates of change, and shading (not visible for γ scale) shows the 90% credible interval from multi-level models fit separately to these data points at each scale. Dashed gray lines show *x* = 0, *y* = 0, and *x* = *y*. (**B**) Kernel density plot of change in β diversity per year calculated as the difference between ∆γ and ∆α (left = homogenization, right = differentiation) of 1000 draws of the posterior distributions of overall mean rates of change at the α and γ scales; the black point shows the median, bar represents 50 (thick) and 90% (thin) credible intervals. Six regions with ∆α > 0.1 (range: 0.1 to 0.3) were removed for clarity from (A); point color represents categories of change from Fig. 1, and points falling on boundaries between categories are gray.

We observed all possible outcomes across the different datasets (Fig. 2A). Many combinations of α and γ scale changes resulted in datasets having no trend in β diversity, many with trends toward homogenization (lower β diversity through time), and many with trends toward differentiation (higher β diversity through time). In total, though, few individual datasets had values of ∆β that statistically differed from zero (fig. S3). Our analysis of all studies combined found a weak mean trend toward homogenization (∆β = −0.003, 90% credible interval (CI): −0.005 to −0.001; Fig. 2B). This change equates approximately to three out of 1000 entirely distinct (i.e., no shared species) communities (*25*) being removed per year (rate of change calculated as the difference between ∆γ and ∆α). Stated differently, this represents an average increase of 0.3% per year in the number of shared species among localities within a region. This mean result was driven by approximately one-half of the datasets that had only two time points, including the checklist data (*n* = 64) and resurvey data (*n* = 232), where contemporary samples were collected to document changes compared to a historical sample (fig. S4). When these data having only two time points were removed from the analysis, we found a slight tendency to differentiation (median ∆β = 0.002, 90% CI: −0.002 to 0.005; fig. S4D). Furthermore, homogenization in the data with only two time points was strongly associated with checklist data (Fig. 2A), and our model showed that residual variation was a decreasing function of sampling duration (fig. S5). This suggests that our observation of a slight tendency toward homogenization in the overall dataset was driven by relatively weak trends at intermediate to large spatial and temporal scales (fig. S6). On the other hand, at smaller to intermediate spatial scales, no change, differentiation, and homogenization are all more common, possibly because at these scales, it is equally plausible that environmental heterogeneity increases, decreases, or does not change at all through time (*9*, *17*).

Although homogenization is often equated with small changes in α diversity, but large declines in γ diversity via larger-scale extinctions (*9*–*11*), we found the opposite result. Our observed weak trend toward homogenization is associated with gains in α diversity more often than losses in γ diversity (Fig. 2A and fig. S3). When α diversity increases more than γ diversity, β diversity goes down. Thus, the weak overall trend to homogenization (∆β < 0) is associated with a small net average increase in α diversity (∆α = 0.004; 90% CI: 0.002 to 0.005), but smaller positive changes in γ scale diversity (∆γ = 0.0006, 90% CI: 0.0003 to 0.0008). Across all datasets, increased numbers of widespread, high occupancy species (Fig. 1i) are a key driver of biotic homogenization (Fig. 2A).

**Fig. 3.**
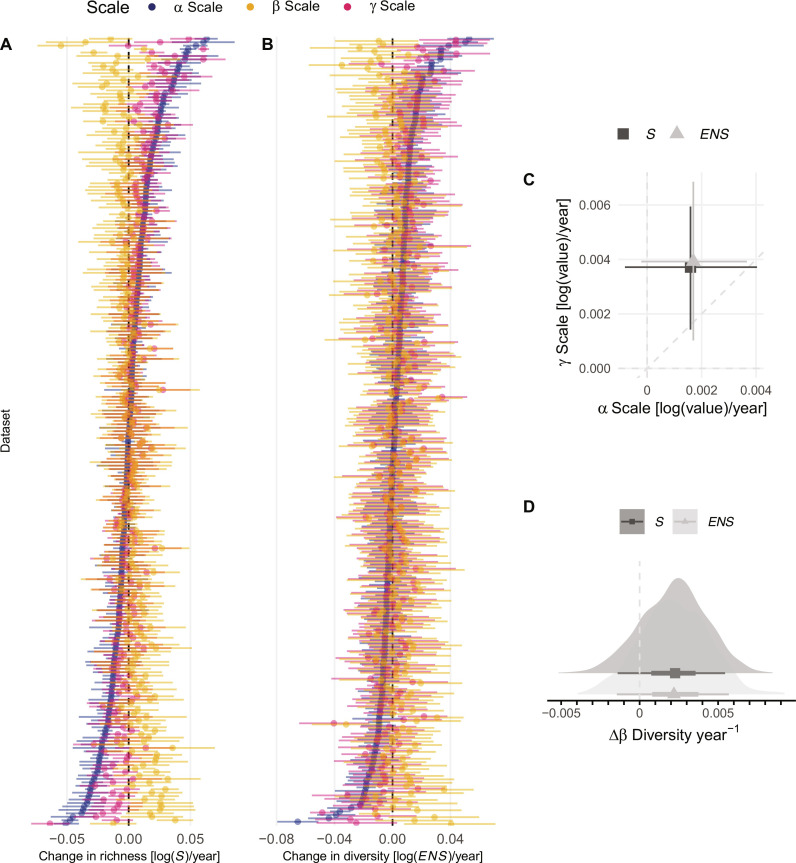
Scale-dependent diversity changes across a subset of 229 metacommunities estimated using models fit to a time series of data. Regional variation in α, β, and γ diversity change estimated using (**A**) species richness (S), (**B**) the effective number of species (*ENS*) conversion of Simpson’s concentration (*25*), (**C**) overall average rates of α and γ scale diversity change, and (**D**) overall estimates of ∆β diversity. Datasets (regions) in (A) and (B) are arranged in order of the magnitude of α diversity changes for each diversity measure [i.e., ordered differently on (A) and (B)]. ∆β Diversity was estimated as the difference between ∆γ and ∆α. Points and whiskers show the median and 90% credible intervals on (A) to (C), as well as the median and 50 and 90% credible intervals on (D).

When we estimated rates of change using models fit to time series data, we again found considerable heterogeneity in scale-dependent diversity changes among regions. Increased power to detect changes, particularly at the γ scale, identified more cases where α and γ scale changes were approximately equal, and multiple cases where ∆γ > ∆α (Fig. 3, A and B). This results in overall average α and γ scale changes being more closely balanced, with ∆γ > ∆α on average (Fig. 3C), meaning average ∆β is slightly increasing (differentiating) but broadly overlapping zero (Fig. 3D; (richness) median = 0.002, 90% CI: −0.001 – 0.005). These results describe changes occurring at smaller to intermediate spatial and temporal scales in comparison to the analysis that included the checklist data in the two time point analyses above and suggest that homogenization and differentiation are approximately balanced at these smaller scales. We also found qualitatively similar results when we used the effective number of species conversion of Simpson’s concentration (which we refer to as *ENS*; Fig. 3B) (*25*). Because species richness is more strongly influenced by rare species, while *ENS* more heavily weights common species (*24*, *25*), we can use any difference in the results to evaluate the role of rare (versus rare and common) species in driving our overall observed patterns. Here, overall results using measures that weight rare species more or less heavily are qualitatively consistent (Fig. 3, C and D), indicating that changes in the numbers and spatial distributions of rare species alone are not the sole driver of observed biodiversity change. Instead, variation in rates of *ENS* change shows that altered numbers of relatively abundant species, either at the α scale of local sites or the larger γ scale, can also drive changes in spatial β diversity (Fig. 3B). Moreover, although temporal changes in richness and diversity are often correlated (*26*), we found the strength of the relationship is considerably weaker at the γ scale compared to the α scale (fig. S7, A and B). This means that a region can be strongly homogenizing or differentiating due to altered numbers and spatial distributions of rare species (reflected in changes in species richness, |∆β_S_| > 0), but not experiencing strong changes in the number and spatial distribution of relatively common species (reflected in *ENS*, ∆β*_ENS_* ~ 0) and vice versa (fig. S7C). This suggests that examining the relationship between changes in the different components of diversity (*26*) across scales will often be required to fully understand drivers of β diversity change.

Although we find some overall tendencies across all of our compiled data (i.e., local gains in species richness slightly outpace regional gains, leading to homogenization, especially for long duration checklist studies), our results primarily indicate that changes in α, β, and γ diversity through time are highly variable across locations, taxa, and time. This matches general findings on local diversity (*4*–*6*, *27*) and population trends (*28*, *29*), where variation in the direction of change means that the strength of overall net trends is weak and is often statistically indistinguishable from zero. We argue that perhaps these results should be expected. There are, of course, many—often anthropogenic—factors that have accelerated in recent decades that could lead to biotic homogenization, including transporting species beyond their historical biogeographic boundaries, and recreating urban or high intensity agricultural landscapes repeatedly (*12*–*14*). However, at the same time, many other impacts could lead to differentiation, including substantial fragmentation of the landscape, the creation of strong spatial gradients of human impact intensity, applying spatially varying resource management practices and land use regulations, and climate change, which induces species to shift at different rates, all leading to spatial heterogeneity (*15*, *16*, *30*). In this context, we stress that the lack of strong overall scale-dependent biodiversity change and prevalence of homogenization should not be taken to indicate that humans are not having a large impact on biodiversity. Both decreases in β diversity, as in homogenization, or increases, as in differentiation, are likely to result from humans modifying nature.

Well-known biases, such as greater sampling effort in the global north when compared to the tropics and Southern Hemisphere, persist in the biodiversity data compiled for this study (fig. S2). This largely limits the generality of our findings to well-studied regions of the planet. In addition, our focus on relating scale-explicit, temporal changes of diversity to altered spatial patterns of species composition (i.e., the number of distinct assemblages within a region) does not track species identities. The extirpation of a species shared among sites in a region matched by a gain of a different shared species has no net effect on the number of distinct assemblages in a region but clearly involves changes that are a key component of biodiversity change (*4*, *5*). Moreover, to parsimoniously link scale-dependent diversity changes to changes in spatial patterns of species composition, we used Whittaker’s (*18*) multiplicative diversity partition [i.e., only two discrete spatial scales (α and γ)] and very simple statistical models. Extensions to examine changes in diversity scaling along a continuum of spatial scales, and to account for sources of nonindependence, such as autocorrelation, and correlated changes in numbers of individuals, species’ relative abundances, and species richness (*26*) are important next steps. Last, extending the framework introduced here to scale-explicit causal attribution of diversity changes promises to strengthen links between biodiversity science and conservation policy (*31*).

Effective conservation is increasingly thought to require protection across multiple sites or at landscape (or larger) spatial scales (*32*, *33*). The simple conceptual typology introduced here simultaneously considers change in α, β, and γ diversity through time, showing how contemporary biodiversity monitoring for management and conservation can embrace a multiscale approach. Here, our results show that different metacommunities are experiencing fundamentally different types of temporal change across scales, resulting in variable trends, and often no trend, in spatial β diversity through time. For metacommunities undergoing change, gains or losses in the number of regionally widespread and/or abundant species can underlie either biotic homogenization or differentiation. This suggests moving beyond a belief of ubiquitous biodiversity loss and homogenization and, instead, working to understand the multiscale nature of biodiversity change and embracing its variability.

## MATERIALS AND METHODS

### Data compilation

Our conceptual typology requires estimates of species richness changes at smaller, local, spatial scales (α diversity) nested within larger, regional, spatial scales (γ diversity). Both scales are somewhat arbitrary and the exact definition varies among data sources. To make our data search and synthesis as comprehensive as possible, we searched broadly for data that met these criteria, where regions had at least four plots or locations, and where richness changes were estimated over a period of at least 10 years. We started by identifying 115 relevant datasets within the BioTIME database (*34*). To this, we added the following: (i) similar assemblage-level time series of studies available in other biodiversity time series compilations not (yet) included in BioTIME [see, e.g., (*35*, *36*)]; (ii) data from studies using “resurveys,” where sites associated with a historical dataset were revisited and re-surveyed using similar methodology in more recent times; (iii) data from “checklist” studies where species known to be present in a given locality (and region) at a “historical” point in time were indicated together with species present in that locality at a later point in time (minus those that went extinct from a site plus those that newly colonized that site); and (iv) data from studies that reported changes in species richness at two spatial scales, but for which the underlying raw data were not available. Because of the relatively specific data requirements and our aim toward a comprehensive growing database, we conducted literature searches and acquired data in an ad hoc fashion by searching multiple relevant databases, rather than using a formal literature search. In all, we compiled a total of 525 regions and a total of 38,166 locations that met our criteria; 461 regions documented repeated samples of species assemblages through time, and 64 regions were compiled from checklist studies (fig. S2 and data file S1).

### Data standardization

To quantify changes in β diversity that emerged from combined changes occurring at the local and regional scale, we required that the start and end years for all locations within a given region were the same. This ensured that change estimated across the different locations within a region covered the same period of time, and meant that regional changes estimated by aggregating all species across all locations within regions also covered the same time period. In addition, to ensure that our analyses did not quantify changes in species richness due to variation in sampling effort, we needed to standardize sampling effort (e.g., the number of plots or transects) across all locations for each time point within regions. The heterogeneous nature of the data that we compiled meant that we needed slightly different procedures to identify combinations of locations and years for different data sources. For clarity, we delineate broad categories of data structures and describe separately how locations and years were selected and sample-effort standardization needed for the different structures.

### Checklist data

Checklist data typically consisted of species lists for locations within regions, compiled from a historical time period and from a more contemporary time period (mean = 251 years; median = 208 years; range = 10 to 518 years). These lists were compiled either from samples and/or observations collected during the two periods or, more frequently, by counting native species only to determine the richness of the historical period, with the contemporary species richness calculated as the sum of native and introduced species (minus any species that went extinct). For our analyses, we selected regions that had at least four locations, removed locations that documented species lists for only one period, and lastly, ensured that all locations within each region had data from the same time period for both the historical and contemporary species lists. Because we were interested in calculating a rate of change, we needed a single date for both the historical and contemporary samples, which in some cases (e.g., oceanic islands) reduces a period of first contact with non-native species to a single time point. Where possible, we used dates from the original data sources. However, where these were not available, approximate dates were estimated for each data source, typically based on when introduced species were likely to have first started arriving, such as first European contact, or post-contact with North America for the flora and fauna of Europe.

### Resurvey data

We distinguish three different data structures that we refer collectively to as resurvey data:

(i) Compiled time series data that document repeated samples of assemblages documenting species abundances, including data from the BioTIME (*34*), RivFishTime (*35*), and InsectChange (*36*), plus similarly structured data from 339 studies that we compiled for this and related studies. We first filtered data to ensure that samples from all locations within regions had a temporal duration of at least 10 years and that a minimum of four locations within a region were sampled per year (allowing us to track the diversity of the same locations through time within regions). Locations within regions were identified using geographic coordinates in the data, although we also included regions with only one geographic coordinate where discrete, unique samples could be identified (e.g., multiple plots within a site).

After applying these filters, the number of locations sampled per year often varied considerably within regions, and we sought to identify locations, as well as start and end years that balanced a trade-off between the number of locations and the duration of the sampling period for each region. To do this, we first identified all year pairs—combinations of start and end year with at least 10 years separating them—for all locations within a given region. We then determined different thresholds for what proportion of the total number of locations we wanted to retain, using a combination of the total number of locations in a region, and visual inspection of locations sampled in each year. For example, for resurvey data newly collated for this study, we selected starting and end years where the proportion of the maximum locations was at least 90% for regions with fewer than 20 locations, 50% for regions with more than 20 locations, and 25% for the NERC Countryside Survey data, which had between 60 and 300 locations across the United Kingdom (and where the lower threshold meant that the temporal duration of the surveys increased by more than 10 years). For regions in the BioTIME and RivFishTime databases, we identified year pairs with at least 75 and 90% of the maximum number of locations, respectively. Multiple year pairs often remained following this, and we selected the pair of years with the longest duration, and lastly, broke any remaining ties by selecting the pair of years with the most locations. For mosquito data sourced from Vectorbase (https://vectorbase.org/vectorbase/app) and compiled in InsectChange, there were frequently fewer sites sampled monthly, and we visually selected locations and the start and end years for each region to ensure that we would be able to standardize the sampling of the same months for each location through time.

Next, we ensured that sampling effort was consistent across all years and locations within regions, using sample-based rarefaction (*37*) where required to standardize effort. Note that for many data (e.g., data from InsectChange and other invertebrate data) where sampling took place across multiple months within years, we used sample-based rarefaction to resample equal numbers of samples across the same months for all locations within a region, which were then compiled to provide one sample per year for each location. In addition, for data collected using multiple sampling methodologies (e.g., mosquitoes sampled using different attractants or freshwater fishes collected with different techniques), we identified the methodology that ensured the maximum number of time series and standardized sampling effort using data collected with only one method.

(ii) We collated data from studies where sites associated with a historical dataset were revisited and resurveyed using the same methodology in more recent times, sometimes referred to as “legacy” studies [see, e.g., (*38*)]. Again, we required each region to have at least four locations and 10 years or more between the historical and contemporary samples.

(iii) Last, we collated studies that estimated species richness changes at two scales, where there were at least four sites at the smaller scale and 10 years between the first and last sample. For these studies, raw data were not available (*n* = 15), but available data provided an estimate of the average local richness at two time points and a single value for regional richness at two time points.

### Estimating richness, diversity, and its change

For the majority of the data, we calculated species richness from the effort-standardized locations and years as the number of distinct species, though higher classifications, such as genera, were sometimes used where studies only classified organisms to genus. We calculated species richness for each location within each region for every available year to document changes in α scale species richness. γ Scale richness was calculated as the number of species in all sites combined for each region and each year. This method of calculating regional richness yields a single number for each region at each time point, and we calculated two types of resamples for regional richness. For datasets where sample-based rarefaction was not required to standardize effort, jackknife resamples were calculated by systematically leaving each location out of the regional richness calculation once. For where effort standardization was more complex and required the use of sample-based rarefaction, we used 200 bootstrap resamples (i.e., richness resamples were estimated using all locations, not *n*_locations_ − 1); then, to prevent these resampled data from dominating the data to which models were fit, we subsampled the bootstrap resamples down to the same size as a jackknife would have been (i.e., we used a random subset of the bootstrap resamples equal to *n*_locations_ – 1 for the given dataset). Last, we summarized both the jackknife and bootstrap resamples by calculating the median regional richness, to which models were fit.

Many data sources, including the 64 datasets with checklist data and 232 regions in the resurvey data, had only 2 years of data available (e.g., a historical and more recent sample). Therefore, to maximize the number of regions in our complete analysis, we calculated richness change using the log ratio of species richness in the most recent time point and species richness in the initial sample, divided by the number of years between the two samples [i.e., $\text{log}\;\left(\frac{S_{t2}}{S_{t1}}\right)t^{-1}$ , where *S*_t2_ is species richness in the most recent sample (t2 = year), *S*_t1_ is species richness in the first sample (t1 = year), and *t* = t2 − t1 + 1 is the number of years between the samples]. This was done separately for each location in each region. These same data were aggregated and used to calculate concomitant changes in regional diversity through time, quantified as the log ratio of resamples (either jackknife or bootstrap) of species richness at the regional scale in the most recent sample and the resample of species richness in the initial sample, divided by the number of years between the two samples.

In addition, because many of our data sources included both information on the abundance of individual species and time series of more than 2 years, we also calculated diversity metrics that differ in their sensitivity to common and rare species (*25*, *39*) for all years having effort-standardized data and estimated rates of change using statistical models. Specifically, we calculated richness and the effective number conversion of Simpson’s concentration (*25*). These two metrics are equal to diversity with order *q* = {0, 2}, where increasing *q* decreases the influence of rare species, and $\mathrm{qD}={\left(\sum_{i=1}^{S},p_{i}^{q}\right)}^{1/\left(1-q\right)}$ ; *p*_i_ is the frequency (relative abundance) of species *i* (*25*). These diversity measures are sometimes referred to as Hill numbers, numbers equivalents, or effective numbers (*25*, *39*), here we refer to them as species richness and *ENS* for simplicity. Moreover, both these measures of diversity can be used in the multiplicative diversity partition of Whittaker (*25*), allowing us to use the one framework overviewed (for the case of species richness) in Fig. 1 to estimate changes at the β scale (∆β) as the difference between changes at the γ and α scales (i.e., ∆γ − ∆α).

### Statistical models

For our first analysis (i.e., rates of richness change calculated using log ratios standardized by time series duration), we estimate α and γ scale richness changes using multilevel (also called mixed effects or hierarchical) models to fit data from each scale separately. For our initial analysis of the changes calculated using log ratios (*n*_regions_ = 525), these models took the form

$${\mathrm{ES}}_{\mathrm{ij}}∼N\left(\mu ,\sigma \right)$$

$$\mu =a+a_{\text{region}\left[i\right]}$$

$$a_{\text{region}}∼N\left(0,\sigma_{\text{region}}\right)$$

$$\text{log}\left(\sigma \right)∼\text{sample}\_{\text{type}}_{i}*\text{log}{\mathrm{dt}}_{i}$$

*ES_ij_* is assumed to have a Gaussian error distribution and is the *j*th local-scale estimate of species richness change in region *i* or, at the regional scale, the *j* subscript is dropped and *ES_i_* is the median of the regional richness resamples for region *i*; *a* is the overall intercept and equal to the average rate of change estimated for each scale, and *a_i_* is the departure from the overall intercept for each region (i.e., the varying intercept for regions) assumed to have a Gaussian distribution with mean zero, and SD estimated from the data. Because variation in the log ratio estimates of change was a decreasing function of the duration between samples, we included covariates for residual variation (σ), where sample_type*_i_* estimated a separate intercept according to whether region *i* were either resurvey or checklist data, and log*dt_i_* was the natural logarithm of the number of years between the first and last samples in region *i*. We fit models using Bayesian methods and we assumed the following,~ weakly regularizing priors

α∼N(0,1)

$$\sigma_{\text{region}}∼N\left(0,1\right)$$

$$\text{sample}\_{\text{type}}_{i}∼N\left(0,1\right)$$

$$\text{log}{\mathrm{dt}}_{i}∼N\left(0,1\right)$$

For the time series data of species richness and the effective number of species conversion of Simpson’s concentration (*n*_regions_ = 229; hereafter *ENS*), we estimated rates of change using statistical models (rather than change being calculated using log ratios as was done in the initial analysis). These time series data were constrained to be positive values but, due to effort standardization, were not always integer values, so we fit models that assumed lognormal error distributions and identity link functions. The models fit to data at the α scale took the form

$$Y_{\mathrm{ij},t}∼\text{lognormal}\left(\mu ,\sigma \right)$$

$$\mu =a+a_{i}+a_{\mathrm{ij}}+\left(b+b_{i}+b_{\mathrm{ij}}\right)x_{\mathrm{ij},t}$$

$$\left[a_{i},b_{i}\right]\prime ∼\text{MVNormal}\left(0,\mathrm{SRS}\right)$$

$$S=\left[\begin{array}{cc} \sigma_{\mathrm{ai}} & 0\\ 0 & \sigma_{\mathrm{bi}} \end{array}\right]$$

$$R=\left[\begin{array}{cc} 1 & \rho_{\mathrm{ai},\mathrm{bi}}\\ \rho_{\mathrm{ai},\mathrm{bi}} & 1 \end{array}\right]$$

$$\left[a_{\mathrm{ij}},b_{\mathrm{ij}}\right]\prime ∼\text{MVNormal}\left(0,\mathrm{SRS}\right)$$

$$S=\left[\begin{array}{cc} \sigma_{\mathrm{aij}} & 0\\ 0 & \sigma_{\mathrm{bij}} \end{array}\right]$$

$$R=\left[\begin{array}{cc} 1 & \rho_{\mathrm{aij},\mathrm{bij}}\\ \rho_{\mathrm{aij},\mathrm{bij}} & 1 \end{array}\right]$$

where *Y_ij,t_* is either species richness or *ENS* of location *j* in region *i* at time *t*; *a* and *b* are the overall intercept and slope (fixed effects), respectively; *a_i_* and *a_ij_* are the departures from the overall intercept for region *i* and location *j* (nested with region *i*), respectively; *b_i_* and *b_ij_* are the departures from the overall slope for region *i* and location *j* (nested with region *i*), respectively; multivariate Gaussian distributions with a mean of zero were assumed for all varying parameters, with the covariance estimated from fitting the model to the data. *x* is time in units of years and was centered by subtracting the mean from all values before model fitting. We assumed weakly regularizing priors. Only the prior for the overall intercept differed between the models for richness and *ENS*, and correlations between levels of the grouping factors are estimated using the Cholesky decomposition of the correlation matrix (**R**), and assumed a Lewandowski-Dorota-Joe (LKJ) prior

a∼N(2,1) for species richness,and

a∼N(1.25,1) for ENS

b∼N(0,0.5)

$$\sigma_{\mathrm{ai}}∼N\left(0,1\right)$$

$$\sigma_{\mathrm{aij}}∼N\left(0,1\right)$$

$$\sigma_{\mathrm{bi}}∼N\left(0,1\right)$$

$$\sigma_{\mathrm{bij}}∼N\left(0,1\right)$$

R∼LKJ(1)

σ∼student_t(3,0,2.5)

The models fit to data at the γ scale took the form

$$Y_{i,t}∼\text{lognormal}\left(\mu ,\sigma \right)$$

$$\mu =a+a_{i}+\left(b+b_{i}\right)x_{i,t}$$

$$\left[a_{i},b_{i}\right]\prime ∼\text{MVNormal}\left(0,\mathrm{SRS}\right)$$

$$S=\left[\begin{array}{cc} \sigma_{\mathrm{ai}} & 0\\ 0 & \sigma_{\mathrm{bi}} \end{array}\right]$$

$$R=\left[\begin{array}{cc} 1 & \rho_{\mathrm{ai},\mathrm{bi}}\\ \rho_{\mathrm{ai},\mathrm{bi}} & 1 \end{array}\right]$$

where *Y_ij,t_* is either species richness or *ENS* of location *j* at time *t*; *a* and *b* are the overall intercept and slope (fixed effects), respectively; *a_i_* are the departures from the overall intercept for region *i*; *b_i_* are the departures from the overall slope for region *i*; multivariate Gaussian distributions with a mean of zero were assumed for all varying parameters, with the covariance estimated from fitting the model to the data. *x* is (mean centered) year. We assumed weakly regularizing priors. Again, only the prior for the overall intercept differed between the models for richness and *ENS*, and correlations between levels of the grouping factors are estimated using the Cholesky decomposition of the correlation matrix (**R**), and assumed an LKJ prior

a∼N(3,1) for species richness,and

a∼N(2,1) for ENS

b∼N(0,1)

$$\sigma_{\mathrm{ai}}∼N\left(0,1\right)$$

$$\sigma_{\mathrm{bi}}∼N\left(0,1\right)$$

R∼LKJ(1)

σ∼N(0,2)

We fit all models using the Hamiltonian Monte Carlo (HMC) sampler Stan (*40*), and models were coded using the brms package (*41*). Models had variable numbers of chains and iterations to ensure posterior distributions had sufficient effective sample sizes. Visual inspection of the HMC chains and Rhat summaries showed model convergence (all Rhats, <1.05).

To quantify proportional changes in β diversity (∆β), we used the models fit to the α and γ scale data (described above), and ∆β was calculated as the difference between ∆γ and ∆α (which are also on a proportional or log scale). Specifically, we calculated the difference between 1000 draws from the posterior distribution of the γ scale estimate (∆γ) and the α scale estimate (∆α). Whittaker’s (*18*) multiplicative partition means the units of our estimate of β diversity are effective numbers of communities (*25*). Variation among regions in ∆β was calculated similarly: estimates of change for individual regions in the initial analysis of log ratios used the varying intercepts (i.e., *a* + *a*_region[*i*]_) and the time series data used the varying slopes for each region (i.e., *b* + *b_i_*), from models fit to data at the α and γ scales. The uncertainty associated with all estimates of change was calculated using draws (at least 1000) from posterior distributions of parameter estimates, and we describe results as statistically significant when the 90% CI does not overlap zero.
