# Supplementary material for: Synthesis reveals approximately balanced biotic differentiation and homogenization
Source: Sci Adv. 2024 Feb 21;10(8):eadj9395. doi: 10.1126/sciadv.adj9395 (PMC10881054; doi:10.1126/sciadv.adj9395)
Supplement: Supplementary file 1 — Figs. S1 to S7 Legend for data file S1 [file sciadv.adj9395_sm.pdf]

Science Advances
AAAS

# Supplementary Materials for

## Synthesis reveals approximately balanced biotic differentiation and homogenization

Shane A. Blowes *et al.*

Corresponding author: Shane A. Blowes, shane.blowes@idiv.de

*Sci. Adv.* **10**, eadj9395 (2024)
DOI: 10.1126/sciadv.adj9395

**The PDF file includes:**

Figs. S1 to S7
Legends for data file S1

**Other Supplementary Material for this manuscript includes the following:**

Data file S1

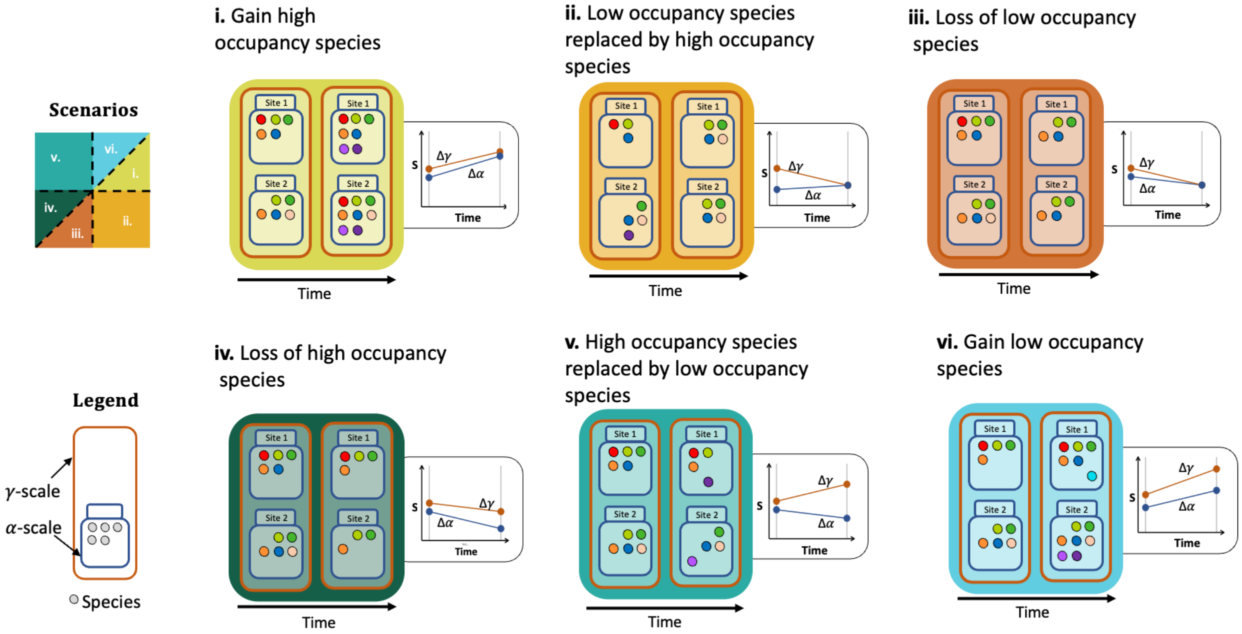


**Figure S1: Simplified illustrations of species occupancy and richness changes underpinning each of the scenarios in Figure 1.** Each panel shows a simplified scenario of richness changes at smaller ($\alpha$) and larger ($\gamma$) scales between two time points, accompanied by a regression showing richness at the two scales as a function time (slopes are labeled $\Delta\alpha$ and $\Delta\gamma$ for each scale).

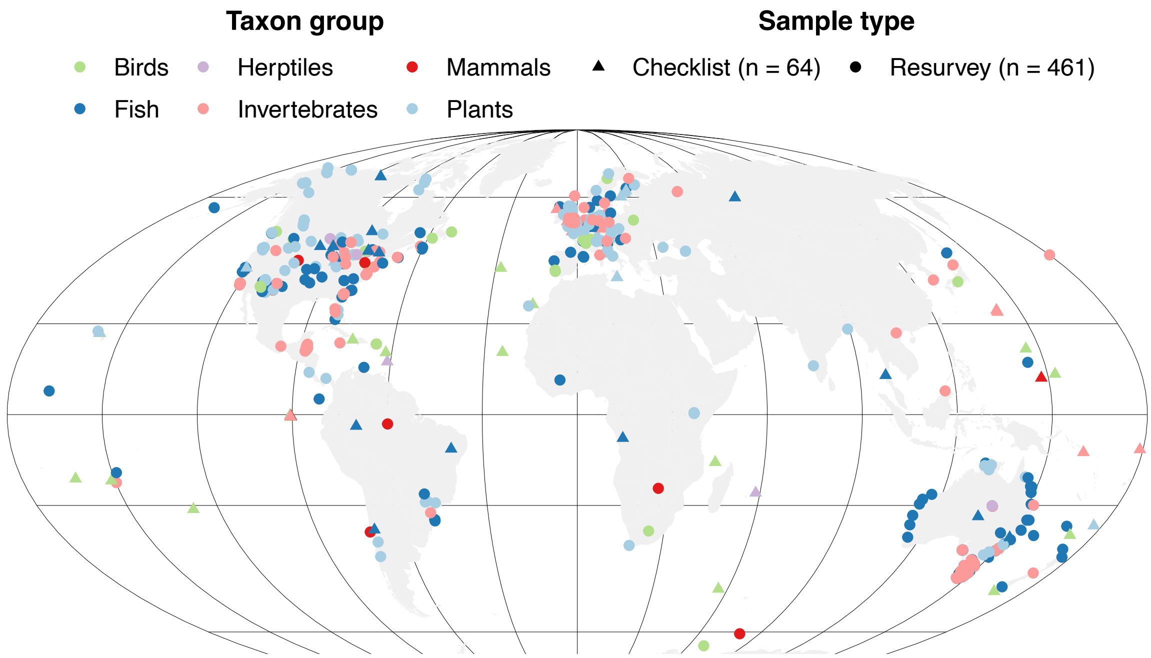


**Figure S2: Map showing the central coordinate of each dataset (region) in our compiled data ($n_{regions}$ = 525).**

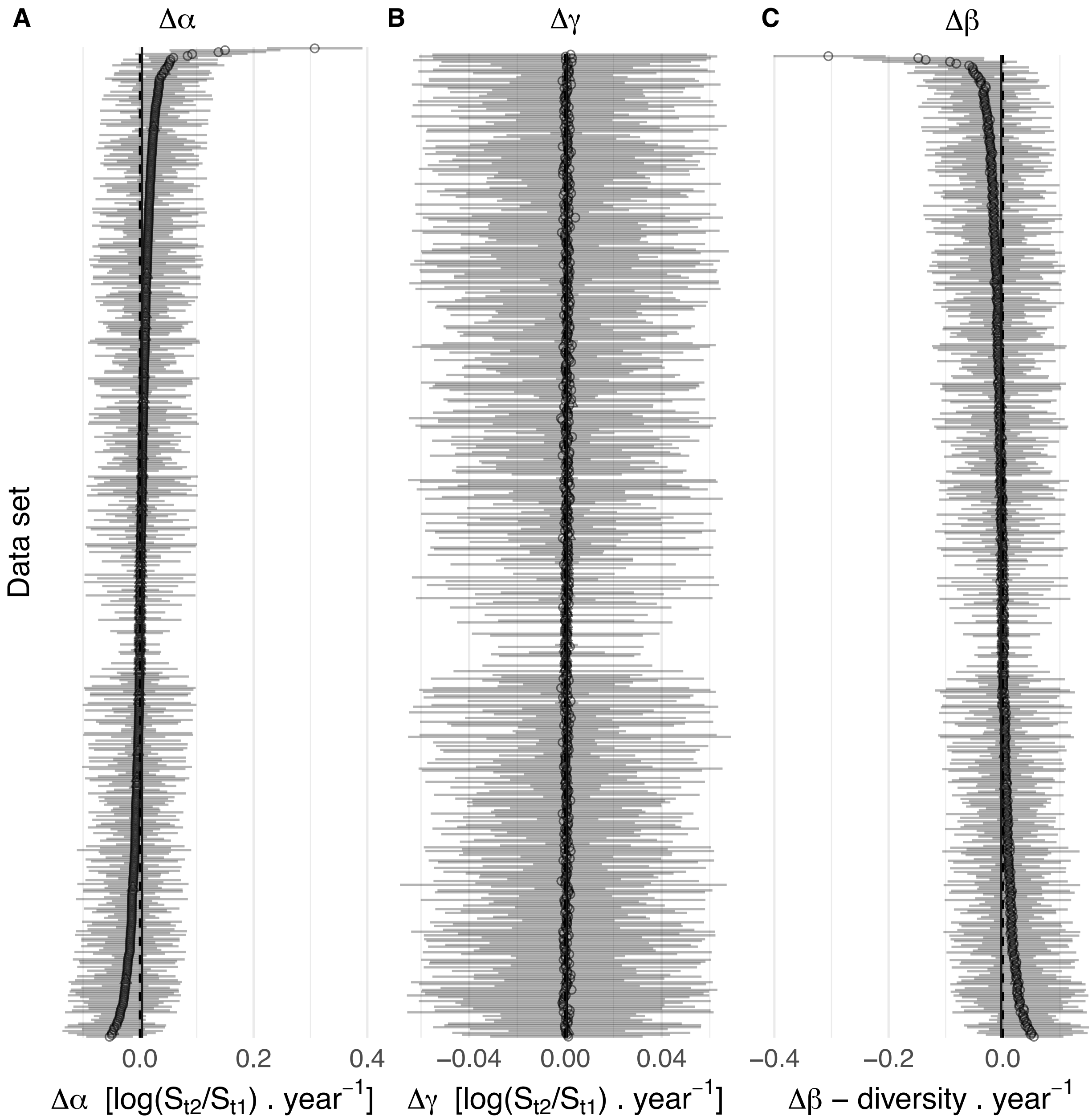


**Figure S3: Model estimates of change for each region (n = 525).** Estimates for changes at the (**A**) $\alpha$-, (**B**) $\gamma$-, and (**C**) $\beta$-scales (change in log[effective number of communities per year]); each point represents a single region, with the bar showing the 90% credible interval; regions are in the same order on panels A-C, arranged by the magnitude of the $\alpha$-scale estimate.

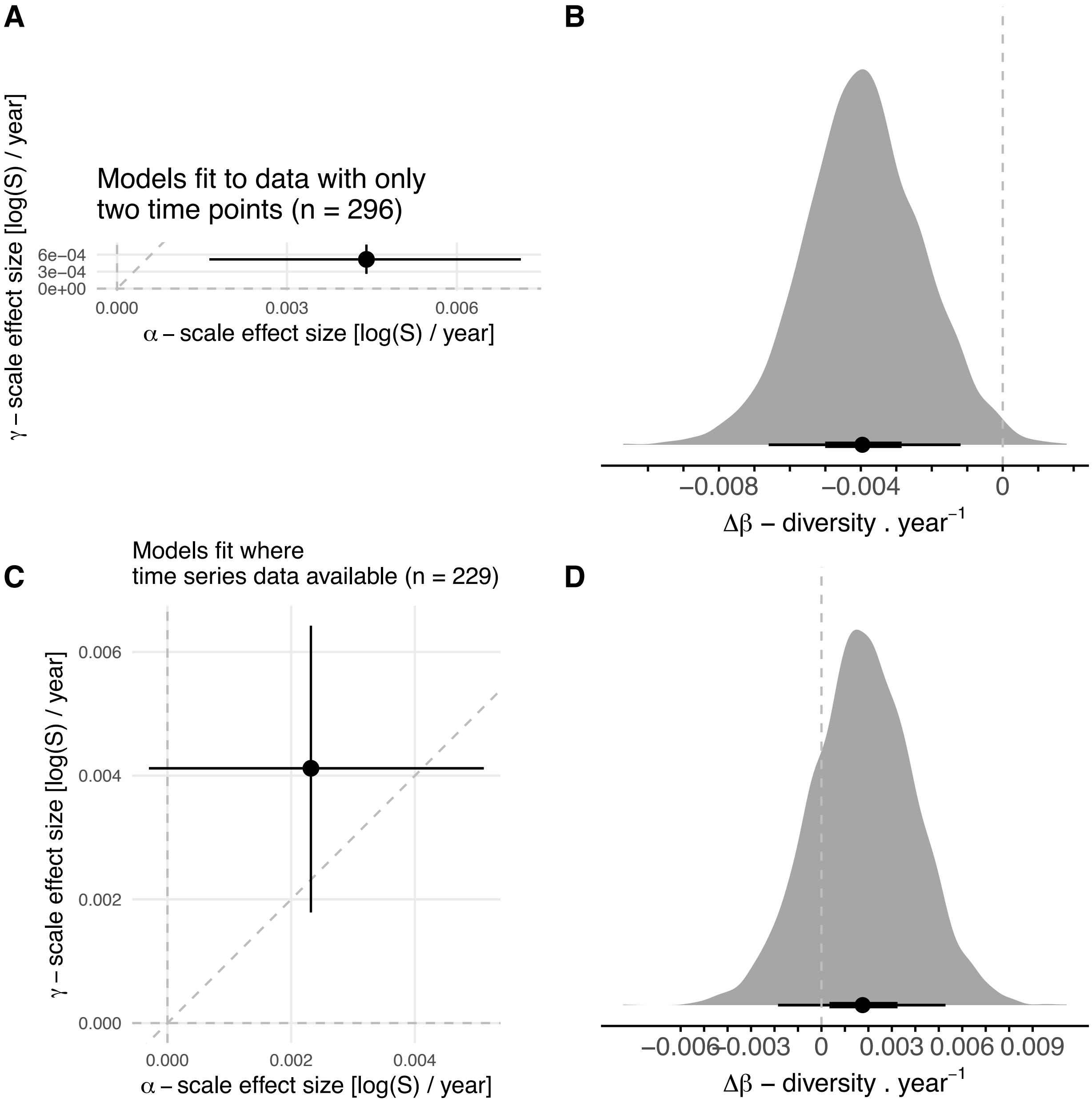


**Figure S4: The tendency towards biotic homogenization is strongest in data where only two time points were available.** (**A**) Change in γ-diversity as a function of change in α-diversity, and (**B**) change in β-diversity estimated by models fit to data with only two time points; (**C**) change in γ-diversity as a function of change in α-diversity, and (**D**) change in β-diversity estimated by models fit to data where time series were available. *Δβ* was calculated as the difference between *Δ*γ and *Δ*α (left = homogenization, right = differentiation) of 1000 draws of *α*- and *γ*-scale intercept posterior distributions; black point shows median, bar represents 50% (thick) and 90% (thin) credible intervals. Note all model results shown here were fit to duration standardized log-ratios as per Fig. 2 in the main text.

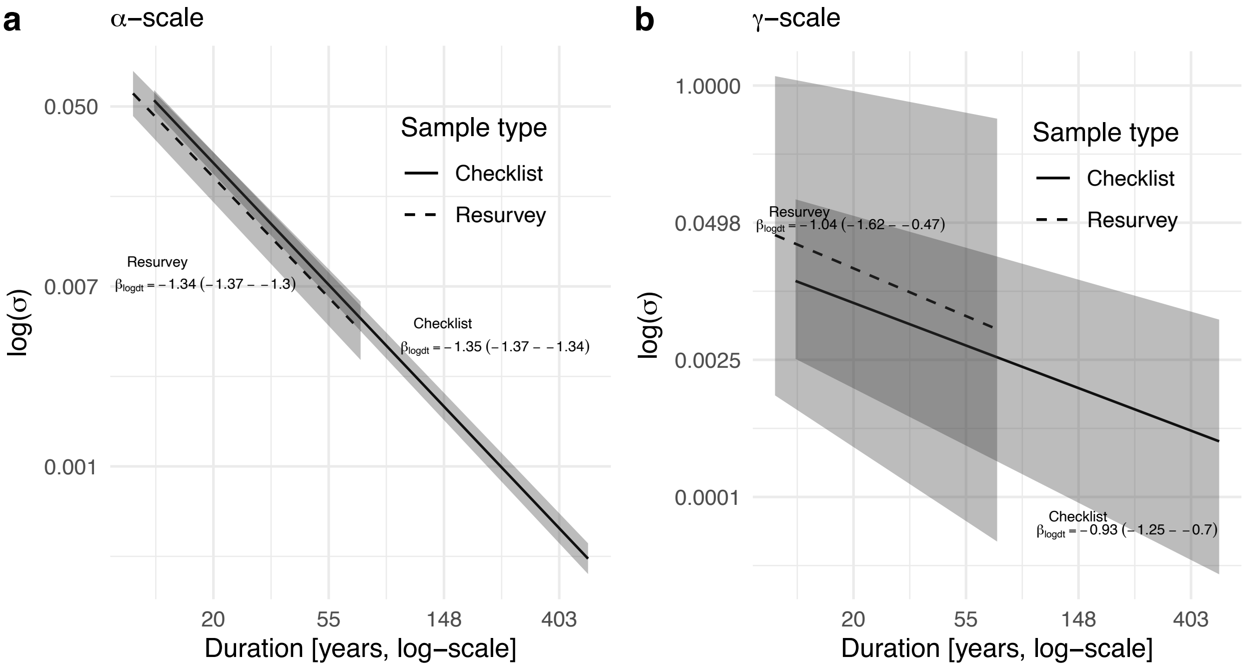


**Figure S5: Residual variation (σ) as a function of temporal duration for the two sample types.** Models fit to the full data set adjusted for residual variation (σ) being a decreasing function of temporal duration for both types of samples (checklists and resurveys) at the (**A**) α- and (**B**) γ-scales.

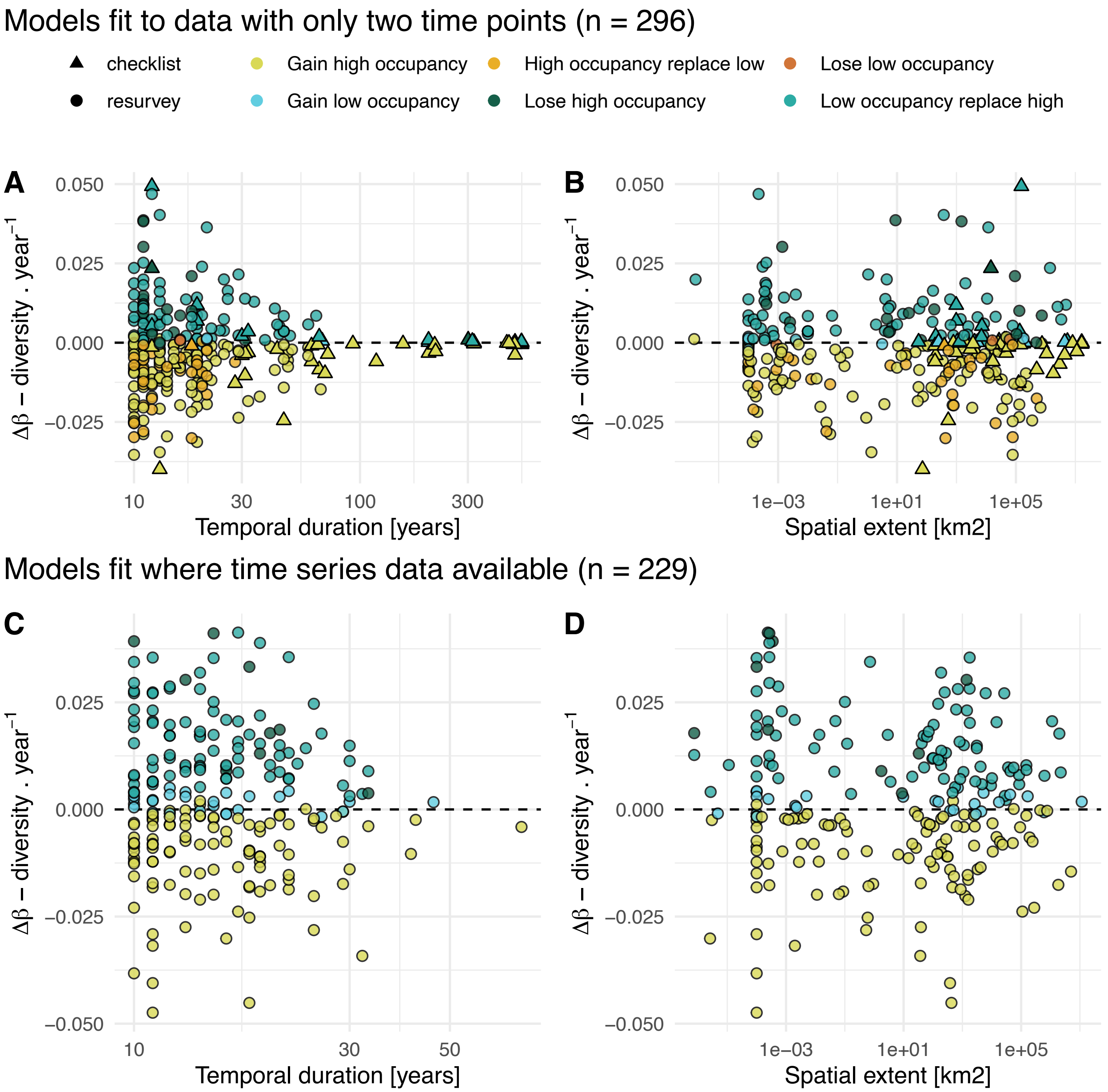


**Figure S6: Biotic homogenization is strongly associated with data sets that had only two time points, and more common at intermediate to large temporal and spatial scales.** Changes in beta-diversity as a function of (**A**) temporal duration and (**B**) spatial extent estimated using models fit to data with only two time points available; and, changes in beta-diversity as a function of (**C**) temporal duration and (**D**) spatial extent estimated using models fit to data where time series were available. Each point shows $\Delta\beta$ for an individual dataset (region) calculated as the difference between $\Delta\gamma$ and $\Delta\alpha$ (< 0 = homogenization, > 0 = differentiation) of 1000 draws of each $\alpha$- and $\gamma$-scale regional estimate (i.e., overall intercept plus regional random intercept). Both x-axes are on a logarithmic scale. To show patterns in the majority of the data more clearly, four regions with $\Delta\beta$ > 0.05 (range: -0.05 - -0.22; duration range: 10-12 years; spatial extent range: 0.002 – 2627km$^2$) were removed from A and B; one region with $\Delta\beta$ = -0.1, duration = 10 years, and spatial extent = 0.0001 km$^2$ was removed from panels C and D.

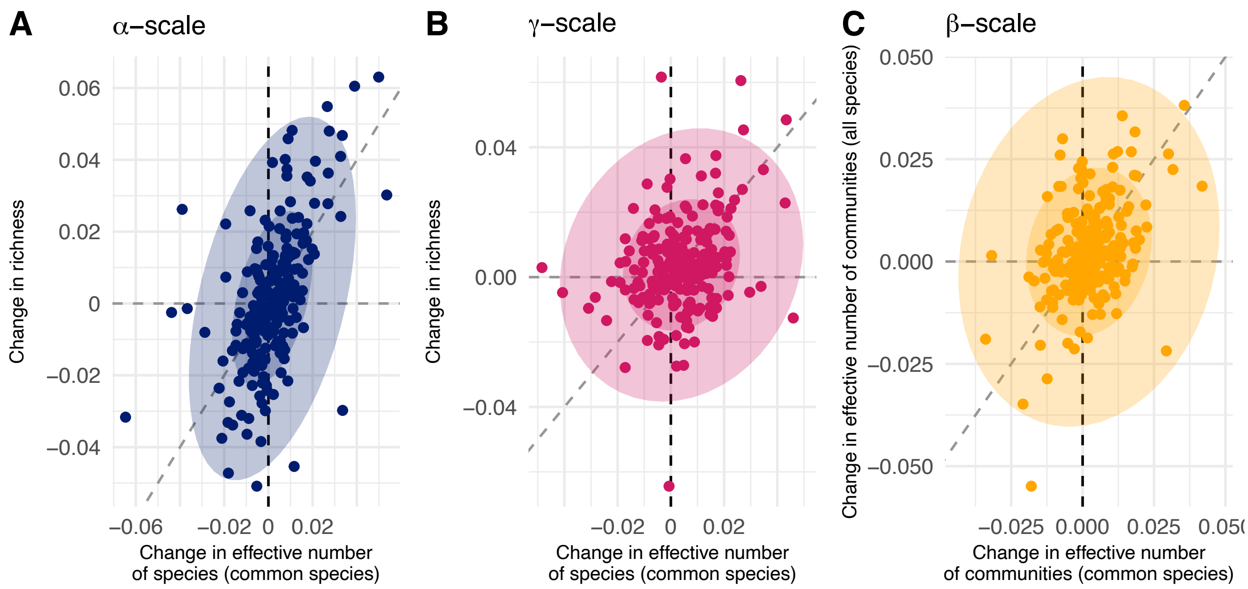


**Figure S7: Relationships between changes in diversity and richness across scales.** Changes in richness as a function of changes in diversity (effective number of species conversion of Simpson's concentration) at the (**A**) α-, and (**B**) γ-scales; (**C**) changes in the effective number of communities for all species as a function of changes in the effective number of communities for common species (i.e., *β*-scale).

**Supplemental data file** (Source data citations.xlsx): Complete citations for the data sources used in the analyses.
